# Optimization of fermentation conditions for cellulase/xylanase production and hydrolysis conditions for efficient conversion of agricultural residues using *Penicillium oxalicum* UNN1

**DOI:** 10.1186/s40643-026-01035-2

**Published:** 2026-03-26

**Authors:** Lingyan Zhong, Fengcheng Jin, Liyuan Qin, Dongping Feng, Weixin Liu, Yuxin Lan, Zhiyun Li, Jiajun Tang, Zhong Cheng, Ting Zhang

**Affiliations:** 1 College of Food and Quality Engineering, Nanning University, Nanning, 530200 Guangxi China; 2 University Engineering Research Center of High-Value Utilization of Tropical and Subtropical Specialty Fruits, Guangxi, Nanning, 530200 Guangxi China

**Keywords:** *Penicillium oxalicum*, Submerged fermentation, Cellulase, Xylanase, Optimization, Hydrolysis

## Abstract

Agricultural residues like sugarcane bagasse and rice straw are rich in cellulose and xylan. Their efficient conversion into (oligo)saccharides and value-added products requires microbial cellulases and xylanases, but low enzyme yields and high production costs hinder industrial application. This study isolated *Penicillium oxalicum* UNN1, a high-xylanase-producing strain with an initial activity of 51.63 U/mL. Submerged fermentation conditions were optimized using different carbon/nitrogen sources to enhance enzyme production. The optimized xylanase activity reached 191.22 U/mL (sugarcane bagasse xylan as sole carbon source) and 142.32 U/mL (combined with Avicel), with filter paper cellulase activity of 0.76 U/mL. The crude enzymes exhibited optimal activity at pH 5.0 and 50 °C. Cellulase retained over 75% activity after 7 h at pH 4.0–6.0 (4 °C) or 40 °C (pH 5.0), while xylanase activity remained nearly unchanged, even after over 21 days of storage at 4 °C (pH 5.0). However, the half-life of xylanase was less than 1 h at 50 °C, though it exceeded 72 h at 40 °C (pH 5.5). 3–5 mM Ca²⁺ and Cu²⁺ strongly inhibited both enzymes. Crude enzyme addition (about 7 U cellulase and 1,400 U xylanase) effectively enhanced reducing sugar production from agricultural residues. Single-factor and response surface optimization yielded optimal hydrolysis conditions: 480 U/g sugarcane bagasse xylan of xylanase, hydrolysate pH of 5.5, hydrolysis temperature of 40 °C, achieving a maximum reducing sugar yield of 0.355 g/g dry biomass. This work demonstrates the potential of *P. oxalicum* UNN1 enzymes for efficient and stable saccharification of agricultural residues, offering a viable approach for their valorization and environmental management.

**Graphical Abstract:**

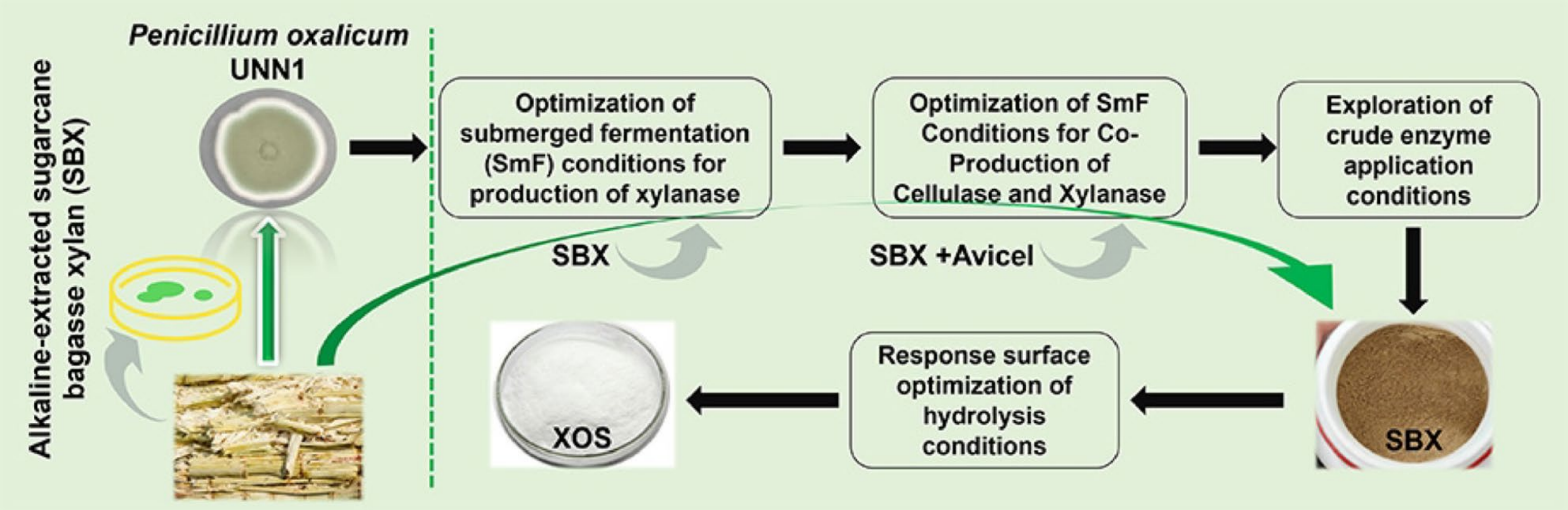

**Supplementary Information:**

The online version contains supplementary material available at 10.1186/s40643-026-01035-2.

## Introduction

Agricultural residues such as sugarcane bagasse and rice straw are generated globally in vast quantities—approximately 5 billion metric tons annually (Bhattacharya et al. 2024). Improper disposal, like open-field burning, causes severe environmental pollution and hinders progress toward the UN Sustainable Development Goals (SDGs) (Bhattacharya et al. 2024). However, these lignocellulosic wastes represent a renewable and abundant feedstock for producing biofuels, biochemicals, and functional oligosaccharides (Lu et al. 2022). Their efficient utilization is crucial for advancing the circular bioeconomy and achieving carbon neutrality.

Lignocellulose has a recalcitrant structure composed of cellulose (β-1,4-glucan), hemicellulose (predominantly β-1,4-xylan), and lignin, which together confer mechanical strength and resistance to biological degradation (Wu et al. 2022). Enzymatic hydrolysis using cellulases and xylanases provides a sustainable and effective strategy to deconstruct this complex polysaccharide matrix into fermentable sugars or prebiotic oligosaccharides (Bueno et al. 2023). The synergistic action of these enzymes is essential for overall saccharification efficiency, as xylanase hydrolyzes the hemicellulose network, reducing physical and chemical barriers and thereby improving cellulose accessibility to cellulases (Chaves De Carvalho et al. 2024).

The global enzymes market is expanding rapidly, projected to reach USD 20.4 billion by 2029 (Adebami et al. 2025), with industrial enzymes accounting for USD 8.42–12.01 billion at a compound annual growth rate (CAGR) of 7.3% from 2025 to 2030 (Industrial enzyme market, 2025; https://www.marketsandmarkets.com/Market-Reports/industrial-enzymes-market-237327836.html). Cellulases and xylanases are among the most widely used industrial enzymes, applied in food processing (Xu et al. 2025) (e.g., brewing (Liu et al. 2024), juice clarification (Bhattacharya et al. 2024), bread improver (Ben Hmad et al. 2024), prebiotic like dietary fiber and oligosaccharides production (Rodriguez et al. 2025; Zhang et al. 2025; Bu et al. 2025), animal feed (Bontà et al. 2024), biofuel production (Meghwanshi et al. 2025), paper and pulp bleaching (Barrios et al. 2025), textiles (Shaikh et al. 2025), detergents (Ajeje et al. 2021), and therapeutic development (Abdelnabi et al. 2025). Despite their broad utility, large-scale application of enzymes is constrained by high production costs, which significantly limit the economic viability of bioprocesses such as lignocellulosic biomass conversion (Vieira et al. 2025). These costs stem from multiple factors, including low enzyme yields, poor stability under operational conditions, limited reusability, and expensive carbon sources (such as beechwood xylan) for fermentation induction (Maurya et al. 2025; Zhang et al. 2025). Therefore, developing bioreactors capable of high-level enzyme production, combined with replacing costly inducers with low-cost, waste-derived substrates like agricultural residues, represents a promising strategy to reduce enzyme costs and advance sustainable biorefining.

In industrial applications, cellulases and xylanases are mainly produced by bacteria, filamentous fungi, and yeasts (Zhao et al. 2024; Šuchová et al. 2022; Khuong et al. 2025). Filamentous fungi, particularly species of the genus *Penicillium*, are known for their strong capacity to secrete lignocellulolytic enzymes (Zhao et al. 2024). Among them, *Penicillium oxalicum* has been reported to produce highly efficient cellulase and xylanase systems, often outperforming *Trichoderma reesei* in certain substrates (Zhao et al. 2024; Wang et al. 2021; Gong et al. 2015). Optimizing fermentation conditions—especially the carbon source for fermentation induction, pH, and temperature—can further enhance enzyme production and tailor the enzyme profile for specific applications (Kabir et al. 2025; Maurya et al. 2025). For example, Maurya et al. (2025) employed Plackett-Burman design (PBD) and Central Composite design (CCD) to optimize the physicochemical parameters for the co-production of xylanase and cellulase by a novel *Pantoea dispersa* in solid-state fermentation. The study revealed that wheat bran, an easily accessible agricultural residue, could serve as a viable alternative to the traditional substrates beechwood xylan and CMC (carboxymethyl cellulose) for the production of xylanase and cellulase. Moreover, optimization using response surface methodology (RSM) could potentially achieve higher production levels (Zhao et al. 2024). Similarly, He et al. (2024) revealed the optimal submerged fermentation (SmF) medium conditions for inducing cellulase production by *T.* reesei RutC-30 using corn stover as an inducer through orthogonal experimental design analysis (He et al. 2024).

In addition, reducing sugars or weakly reducing oligosaccharides are important raw materials in the chemical industry and the functional prebiotics field. For example, glucose and xylose can be used in fermentation processes to produce biofuels and other biochemicals (Kwak et al. 2019). Oligosaccharides such as xylo-oligosaccharides (XOS) and arabinoxylo-oligosaccharides (AXOS) can help regulate specific probiotic species in the gut, promoting intestinal health (Huang et al. 2025). Therefore, optimizing the hydrolysis conditions of agricultural residue or its derived polysaccharides can enhance the yield of these valuable enzymatic products, thereby reducing the cost of biotransformation feedstocks.

In this study, we isolated a novel *P. oxalicum* strain UNN1 exhibiting high xylanase productivity. We optimized its SmF conditions using agricultural waste-derived carbon sources to maximize both xylanase and cellulase yields. The biochemical properties of the crude enzymes, including pH and thermal stability, metal ion effects, and hydrolytic efficiency on sugarcane bagasse, were systematically evaluated. Furthermore, single-factor and RSM were employed to optimize the enzymatic hydrolysis conditions for the potential production of reducing sugars and possible oligosaccharides. This work aims to develop an efficient and stable enzyme system for the valorization of agricultural residues, providing technical support for sustainable biomanufacturing and pollution mitigation.

## Materials and methods

### Microorganism strains, main materials, and culture conditions

Fungal strains exhibiting xylanase activity were isolated from sugarcane field soil in Guangxi, China, using a screening medium containing 10 g/L sugarcane bagasse xylan (alkaline-extracted, SBX; Fig. S1). All strains were cryopreserved at -80 °C in 25% glycerol (Sangon Biotech [Shanghai] Co. Ltd., Shanghai, China) for permanent storage, and for short-term storage, PDA medium (potato dextrose agar; Becton, Dickinson and Company, Sparks, MD, USA) was used, which was also employed for generating asexual spores of the strains by cultivating them for 5 days at 28 °C. Spore suspensions were prepared by dispersing conidia of fungal strains from PDA medium into 0.2% (v/v) Tween-80 (Sangon Biotech) and standardizing to 1 × 10^8^ spores·mL^− 1^. The strain *Penicillium oxalicum* UNN1 (CCTCC AF 2025015) was identified through ITS sequencing (GenBank PX588462) (Feng et al. 2024).

For the preparation of crude enzyme solutions from SmF of the fungal strains, a fresh spore suspension containing 1 × 10^8^ spores was inoculated into the initial liquid fermentation medium, containing 10 g/L inducible carbon source, 4 g/L (NH4)_2_SO_4_, 4 g/L KH_2_PO_4_, 0.6 g/L MgSO_4_·7H_2_O, 0.6 g/L CaCl_2_, 1 mL/L Tween-80, and 100 µL/L trace element mixture (Zhang et al. 2021). The medium was adjusted to pH 5.5 and sterilized by moist heat at 121 °C for 20 min before inoculation. Subsequently, the mixture was incubated in a shaking incubator at 28 °C and 180 rpm for 120 h. Upon completion of fermentation, the mixture was centrifuged at 12,000× g for 10 min at 4 °C. The supernatant was collected and used directly as the crude enzyme solution for subsequent enzyme activity assays and analyses.

SBX was prepared as an inducible carbon source following a chemical extraction procedure. Briefly, sugarcane bagasse was dried at 60 °C and ground to pass through an 80-mesh sieve. The resulting powder was mixed with 10% (w/v) KOH solution at a solid-to-liquid ratio of 1:10 (w/v), and the mixture was incubated in a shaking incubator at 40 °C and 200 rpm for 20 h. After reaction, the mixture was centrifuged at 12,000× g for 10 min. The supernatant was collected, and its pH was adjusted to 6.5 with glacial acetic acid. Xylan was then precipitated by adding four volumes of anhydrous ethanol, followed by incubation at 4 °C for 2 h. The precipitate was recovered by centrifugation at 12,000× g for 10 min, and then dried to constant weight at 60 °C. The purified SBX was ground into powder and stored in a sealed container under cool and dry conditions for subsequent use.

In addition, common chemical reagents such as 3,5-dinitrosalicylic acid, (NH4)_2_SO_4_, and KOH were of analytical grade. Agricultural residues, including sugarcane bagasse, rice straw, and so on, were collected from local farmers.

### Determination of the cellulase and xylanase activity

Cellulase/xylanase activities were determined using the 3,5-dinitrosalicylic acid (DNS) method with slight modifications to the protocol described previously (Zhang et al. 2023). The DNS reagent was prepared by dissolving 10 g/L 3,5-dinitrosalicylic acid, 20 g/L NaOH, 0.5 g/L sodium sulfite, and 200 g/L C_4_H_4_KNaO_6_·4H_2_O, followed by the addition of 2 g/L phenol. The solution was stirred thoroughly, filtered to remove insoluble impurities, and stored at room temperature in the dark for 7 days before use. Enzyme activity was measured based on the amount of reducing sugars released from the appropriate substrate under standard assay conditions. In brief, 500 µL or 50 µL of diluted enzyme solution was mixed with 1,500 µL or 450 µL of 50 mM citrate-Na_2_HPO_4_ buffer (pH 5.0), containing a piece of Whatman No. 1 filter paper (1 × 6 cm; GE Healthcare Life Sciences, UK) and 10 g/L beechwood xylan (purity = 90%, Shanghai Yuanye Biotechnology Co., Ltd.), respectively. The reaction was carried out by incubating the enzyme-substrate mixture at 50 °C for 60 min (cellulase) or 10 min (xylanase). The reaction was stopped by adding 3 mL or 1 mL of DNS reagent, respectively. After centrifugation at 12,000× g for 1 min, an equal volume of supernatant was heated in boiling water for 5 min, then cooled on ice and brought to room temperature. Absorbance at 540 nm was measured using a spectrophotometer (SpectraMax ABS Plus, USA) against a glucose (cellulase) or xylose (xylanase) standard curve. One unit (U) of enzyme activity was defined as the amount that releases 1 µmol of reducing sugar (as glucose or xylose equivalents) per minute under the specified assay conditions. In this study, U/mL—used throughout the SmF-condition optimization—denotes the catalytic activity in the crude enzyme solution, while U/g SBX—applied during the hydrolysis-condition optimization—indicates the enzyme loading per gram of substrate SBX. All assays were conducted at least in triplicate to ensure reproducibility.

### Single-factor optimization of SmF conditions for enzyme production

This study optimized the fermentation of *P. oxalicum* UNN1 through single-factor experiments, focusing on medium composition (carbon and nitrogen sources) and environmental conditions (fermentation time, temperature, agitation speed, and initial medium pH). For the optimization of fermentation conditions using SBX as the sole carbon source, the following factors were investigated in a single-factor, multi-level sequential optimization manner: the concentration of SBX (5 g/L, 10 g/L, 15 g/L, 20 g/L, 25 g/L, 30 g/L, 35 g/L, 40 g/L, 45 g/L, and 50 g/L), types of nitrogen sources (4 g/L NaNO_3_, (NH4)_2_SO_4_, peptone, urea, yeast extract, soy peptone), fermentation time (72 h, 96 h, 120 h, 144 h, 168 h), fermentation temperature (24 °C, 26 °C, 28 °C, 30 °C, 32 °C), agitation speed (140 rpm, 160 rpm, 180 rpm, 200 rpm, 220 rpm), and initial medium pH (4.5, 5.0, 5.5, 6.0, 6.5).

For the optimization of fermentation conditions using a combination of SBX and Avicel as carbon sources, the following factors were optimized in a single-factor, five-level sequential manner: the ratio of SBX to Avicel (1:4, 2:3, 1:1, 3:2, 4:1; 10 g/L total carbon source), types of nitrogen sources (4 g/L NaNO_3_, (NH4)_2_SO_4_, peptone, urea, yeast extract, soy peptone), initial medium pH (4.5, 5.0, 5.5, 6.0, 6.5), fermentation time (72 h, 96 h, 120 h, 144 h, 168 h), fermentation temperature (24 °C, 26 °C, 28 °C, 30 °C, 32 °C), and agitation speed (140 rpm, 160 rpm, 180 rpm, 200 rpm, 220 rpm).

### Impact of pH and temperature on cellulase and xylanase activity and stability

After obtaining the crude enzyme solution under the optimal enzyme production conditions using a combination of SBX and Avicel as carbon sources, the enzymatic properties and stability of the crude cellulase and xylanase were investigated. Initially, the effects of different pH values (3.0, 4.0, 5.0, 6.0, 7.0) on the activity of filter paper cellulase (FPase, used to characterize cellulase) and xylanase were examined (Sect.  2 of Materials and Methods). Concurrently, the impact of reaction temperature (30 °C, 40 °C, 50 °C, 60 °C, 70 °C) on enzyme activity was studied, with the substrate solution prepared using the optimal reaction pH buffer, and pH/temperature-relative enzyme activity curves were plotted.

Subsequently, the stability of the crude enzyme at different pH levels was assessed. The crude enzyme solution was diluted with 50 mM citrate-Na_2_HPO_4_ buffer (pH = 4.0, 5.0, 6.0) and incubated at 4 °C for 1–7 h. Samples were taken at 1 h, 2 h, 4 h, and 7 h for enzyme activity measurement and relative activity calculation, with the enzyme activity of the solution incubated for 0 h set as 100%. Regarding thermal stability, the crude enzyme, diluted with the optimal reaction pH buffer, was incubated at 40 °C, 50 °C, and 60 °C for 1 h, 2 h, 4 h, and 7 h, and the enzyme activity was measured to evaluate the thermal stability of the crude enzyme. Additionally, the enzyme activity was determined after extended incubation for 21 days at 4 °C (pH = 5.0) to assess storage stability, with the enzyme activity of the solution incubated for 0 h at different temperatures set as 100%. Referring to the hydrolysis temperature in the published literature by Zhao et al. (2021) and the temperature stability test results of the crude enzyme in this study, the half-life of the crude xylanase was further determined under the condition of 40 °C (pH = 5.5) to establish the enzymatic hydrolysis time range for subsequent enzymatic hydrolysis experiments.

### Metal ions’ effects on cellulase and xylanase activity

To determine the effects of common metal ions found in industrial production on the activity of the crude enzyme, various metal ions (Ca^2+^, Ba^2+^, Na^+^, K^+^, Mn^2+^, Mg^2+^, Fe^2+^, Co^2+^, Cu^2+^, Zn^2+^; all as chlorides) were added to the crude enzyme solution diluted with a pH 5.0 buffer. The final concentrations of each metal ion were set to 3 mM and 5 mM. After reacting at room temperature (approximately 25 °C) for 30 min, the activities of the FPase and xylanase were measured, and relative activities were calculated, with the enzyme activity of the solution without added metal ions set as 100%.

### Enzymatic hydrolysis of agricultural residues

Agricultural residues such as peanut shells, corn cobs, orange peels, pomelo peels, rice straws, wheat bran, and sugarcane bagasse (rich in cellulose and xylan) were pre-treated. Initially, these residues were dried to constant weight at 60 °C. Subsequently, the dried residues were ground and passed through a 40-mesh sieve to obtain uniform particles. A weighed amount of 0.5 g of the treated substrate was mixed thoroughly with 40 mL of the optimized SmF medium (with SBX plus Avicel as the combined carbon source). To each sample, 10 mL of the crude enzyme solution (containing approximately 7 U of FPase and 1400 U of xylanase) was added. The mixtures were hydrolyzed at 40 °C and 180 rpm for 48 h (with sampling every 12 h). After hydrolysis, the samples were centrifuged at 12,000× g for 10 min, and the concentration of reducing sugars in the supernatants was determined using the DNS method (expressed as glucose equivalents). Briefly, 500 µL of appropriately diluted supernatant was mixed with 1 mL of DNS reagent, heated in a boiling water bath for 5 min, rapidly cooled in an ice bath, and then brought back to room temperature before measuring the absorbance at OD_540_. The concentration of reducing sugars (g/L) in the samples was calculated according to the glucose standard curve. The reducing sugar yield (g/g dry biomass = g/L*0.05 L/0.5 g dry biomass) was subsequently expressed as grams of reducing sugars per gram of dry biomass (g/g dry biomass) based on the initial dry weight of the substrate.

### Enzymatic hydrolysis of SBX and optimization of hydrolysis conditions by single-factor and response surface methodology

To explore the ability of the crude enzyme to hydrolyze SBX for the production of reducing sugars or weakly reducing prebiotic xylooligosaccharides, this study conducted an enzymatic hydrolysis experiment of SBX. Initially, 1 × 10^8^ spores of strain UNN1 were inoculated into the optimal SmF medium with 25 g/L SBX as the sole carbon source for xylanase production and cultured at 28 °C and 200 rpm for 120 h. The xylanase activity in the crude enzyme solution was measured, and the solution was then sterilized by filtration through a 0.22 μm hydrophobic membrane (Millipore, Germany) and stored at 4 °C for later use. The pre-optimization hydrolysis conditions were as follows: 480 U/g SBX of xylanase was added to the hydrolysis medium containing 10 g/L SBX (the SmF medium optimized in Sect.  3 of Materials and Methods, pH = 5.5), and the mixture was cultured in a shaking incubator at 40 °C and 180 rpm for a specific duration.

Three factors at three levels were selected for single-factor and response surface optimization: xylanase dosage (240 U/g, 480 U/g, and 720 U/g SBX), pH of hydrolysate (5.0, 5.5, 6.0), and hydrolysis temperature (30 °C, 40 °C, 50 °C). The yield of reducing sugars was used as the detection index. Samples were taken at 0 h, 2 h, 4 h, 12 h, 24 h, and 48 h, centrifuged at 12,000× g for 10 min, and the reducing sugar concentration (g/L) was measured using the same method as in Sect.  2 of Materials and methods, except that the calculation was based on the xylose standard curve to determine the reducing sugar content in the samples (expressed as xylose equivalents). The reducing sugar yield (g/g SBX = g/L*0.05 L/0.5 g SBX) was subsequently expressed as grams of reducing sugars per gram of SBX (g/g SBX) based on the initial dry weight of the substrate. The experimental design of the response surface optimization and the measurement results of reducing sugar yield are shown in Table 1.

**Table 1:** Response surface design and results for the optimization of reducing sugar concentration

Std	Factor 1 A: Xylanase dosage (U/g SBX)	Factor 2 B: pH of hydrolysate	Factor 3 C: Hydrolysis temperature (°C)	Response 1 Reducing sugar yield (g/g SBX)
1	240	5.0	40	0.3162
2	720	5.0	40	0.2944
3	240	6.0	40	0.3080
4	720	6.0	40	0.3024
5	240	5.5	30	0.3151
6	720	5.5	30	0.3313
7	240	5.5	50	0.3215
8	720	5.5	50	0.3333
9	480	5.0	30	0.3005
10	480	6.0	30	0.3387
11	480	5.0	50	0.3300
12	480	6.0	50	0.3268
13	480	5.5	40	0.3663
14	480	5.5	40	0.3633
15	480	5.5	40	0.3576
16	480	5.5	40	0.3680
17	480	5.5	40	0.3760

### Statistical analysis

All experimental measurements were performed in triplicate, and the resulting data were analyzed statistically using one-way analysis of variance (ANOVA) with a significance level of *p* ≤ 0.05, carried out in SPSS 27 (IBM, Armonk, NY, USA). Where appropriate, differences between two groups were assessed using Student’s *t*-test with Microsoft Office 2016 (Microsoft, Redmond, WA, USA). For the molecular dynamics simulation, we used the software GraphPad Prism version 10.1.2. For optimization and modeling of the experimental parameters, a Box-Behnken design combined with RSM was employed, and data were analyzed using Design Expert 13.0 software.

## Results and discussion

### Optimization of SmF conditions for production of xylanase by *P. oxalicum* strain UNN1

**Fig. 1:**
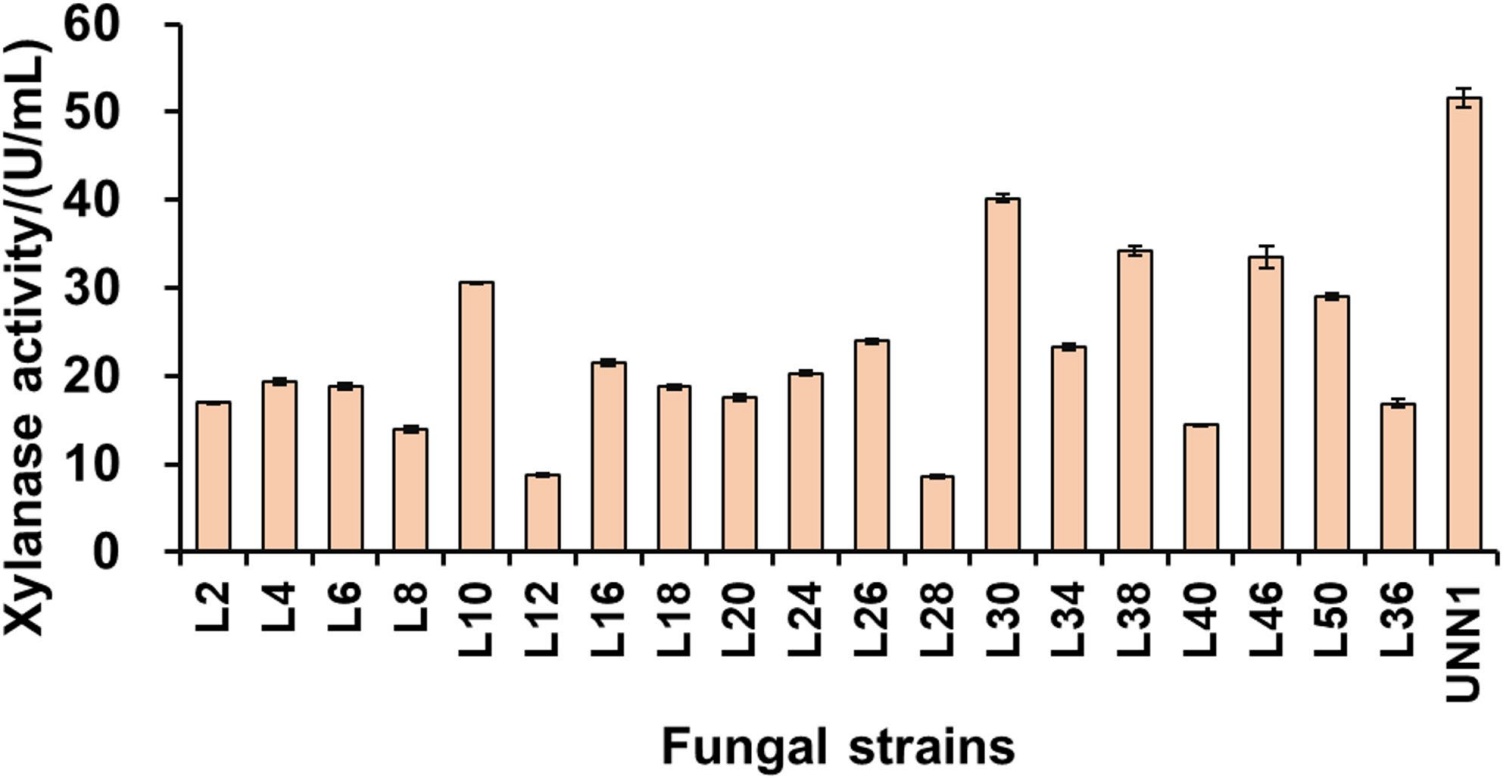
Xylanase activity assay of 20 fungal strains. All strains were cultured in a liquid medium containing 10 g/L of beechwood xylan for 120 h. The results presented are the means ± standard deviation (SD)

**Fig. 2:**
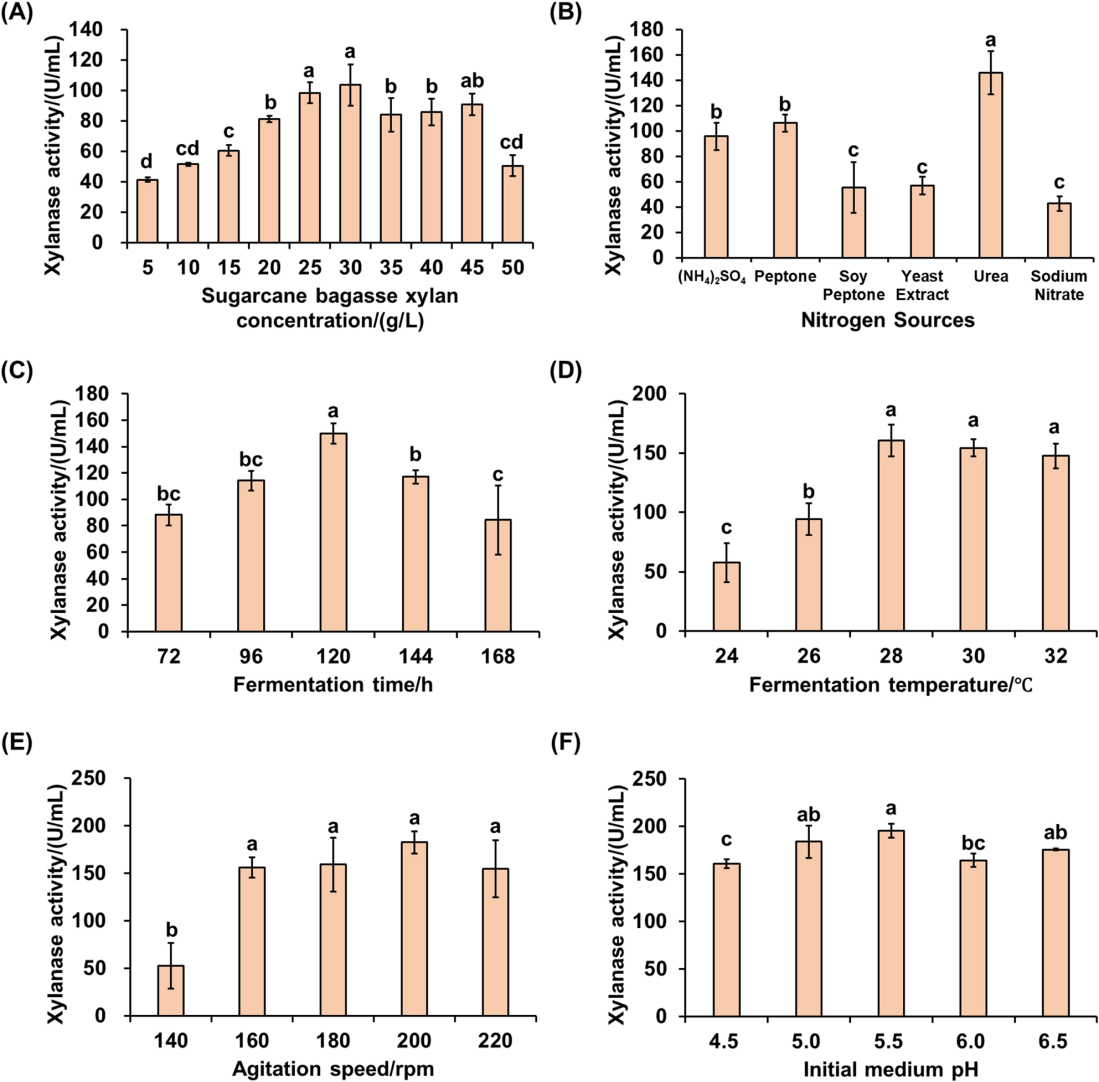
Sequential optimization of xylanase production by *P. oxalicum* strain UNN1. **A** Effect of alkaline-extracted sugarcane bagasse xylan concentration (g/L) on xylanase activity. **B** Effect of types of nitrogen sources at 4 g/L on xylanase activity. **C** Effect of fermentation time (h) on xylanase activity. **D** Effect of fermentation temperature (°C) on xylanase activity. **E** Effect of agitation speed (rpm) on xylanase activity. (F) Effect of the initial medium pH on xylanase activity. Data are presented as mean ± SD (*n* = 3), bars marked with different letters indicate significant differences (*P* < 0.05)

From the soil of sugarcane fields in Guangxi, we successfully identified 20 fungal strains that exhibit xylanase activity (Fig. S1). Among these strains, UNN1 demonstrated the highest xylanase activity, with a yield of approximately (51.63 ± 1.06) U/mL after 120 h of cultivation (Fig. 1). In this study, the conditions for xylanase production were optimized sequentially, including the concentration of alkaline-extracted SBX (as the sole carbon source), types of nitrogen sources, fermentation time, fermentation temperature, agitation speed, and initial medium pH. The optimal parameters were determined as follows: 25 g/L alkaline-extracted SBX, yielding (98.41 ± 6.74) U/mL of xylanase (Fig. 2A); 4 g/L urea, yielding (145.91 ± 16.96) U/mL of xylanase (Fig. 2B); a fermentation time of 120 h, yielding (150.05 ± 7.74) U/mL of xylanase (Fig. 2C); a fermentation temperature of 28 °C, yielding (160.67 ± 13.41) U/mL of xylanase (Fig. 2D); an agitation speed of 200 rpm, yielding (182.52 ± 11.66) U/mL of xylanase (Fig. 2E); and an initial medium pH of 5.5, yielding (195.35 ± 7.20) U/mL of xylanase (Fig. 2F). Under the optimized parameters, as verified, the xylanase activity reached (191.22 ± 4.27) U/mL, representing a 2.84-fold increase compared with the initial activity of (49.81 ± 0.52) U/mL. It is worth noting that different nitrogen sources have a significant impact on the xylanase production of the strain, highlighting the importance of selecting the appropriate nitrogen source for industrial applications. Moreover, lower fermentation temperatures (24–26 °C) and agitation speeds (140 rpm) severely inhibit the enzyme production efficiency of strain UNN1, indicating that strain UNN1 is sensitive to environmental conditions.

These results further demonstrate that strain UNN1 is a promising candidate for xylanase production. As shown in Table 2, the xylanase activity achieved here using alkaline-extracted SBX as the sole inducer (191.22 U/mL) compares favorably with, or exceeds, that of other *P. oxalicum* strains grown on various substrates, including commercial xylan (e.g., *P. oxalicum* 5–18, 30.83 U/mL) (Hu et al. 2023) and complex agricultural residues (e.g., *P. oxalicum* GZ-2, 115.2 U/mL) (Liao et al. 2012). More importantly, replacing commercial beechwood xylan with agricultural residues and their derivatives as the inducer may reduce the carbon source cost, which constitutes a core economic advantage of this technology. Although precise production costs at the ton-scale require pilot-scale verification, the cost reduction from feedstock substitution indicates its application potential. Building upon these foreseeable advantages and considering the cost-effectiveness in practical industrial applications, future research should focus on maintaining high enzyme yields while further optimizing the process through systematic approaches such as RSM, thereby achieving an even more economically efficient production system.

**Table 2:** Comparative analysis of cellulase and xylanase production by different *Penicillium oxalicum* strains

Strain	Substrate	Fermentation mode	Enzyme activities	References
*Penicillium oxalicum* UNN1	2.5% (w/v) sugarcane bagasse xylan (SBX)	submerged fermentation (SmF)	(191.22 ± 4.27) U/mL (xylanase)	this study
*P. oxalicum* UNN1	SBX: Avicel = 3:2 (w/w; 10 g/L)	SmF	(0.76 ± 0.03) U/mL (Filter paper cellulase; FPase) and (142.32 ± 9.19) U/mL (xylanase)	this study
*P. oxalicum* 5–18	1% C xylan	SmF	30.83 U/mL (xylanase)	Hu et al. 2023
*P. oxalicum* GZ-2	2% (w/v) wheat straw	SmF	115.2 U/mL (xylanase)	Liao et al. 2012
*P. oxalicum* IODBF-5	0.5% (w/v) Avicel and 2.5% (w/v) wheat bran	SmF	1.26 U/mL (FPase)	Saini et al. 2015
*P. oxalicum* RE-10	20 g/L DCCR, 6 g/L MCC, 46.5 g/L WB, 10 g/L soybean cake powder	SmF (7.5-liter fermenter)	17.66 U/mL (FPase)	Han et al. 2017a
*P. oxalicum* RE-10	20 g/L delignined corn cob residue from xylitol production (DCCR), 6 g/L microcrystalline cellulose (MCC), 45.58 g/L wheat bran (WB)	SmF	8.61 U/mL (FPase)	Han et al. 2017b
*P. oxalicum* OE-CX^C^-S-1	2% corn cob residue, 0.6% microcrystalline cellulose, 4.66% wheat bran, 1.0% soybean cake powder	SmF	2.8 U/mL (FPase)	Gao et al. 2017
*P. oxalicum* G2	1.5% (w/v) radix isatidis residues (RIR)	SmF	2.2 U/mL (FPase)	Zhang et al. 2019

### Optimization of SmF conditions for co-production of cellulase and xylanase by *P. oxalicum* strain UNN1

**Fig. 3:**
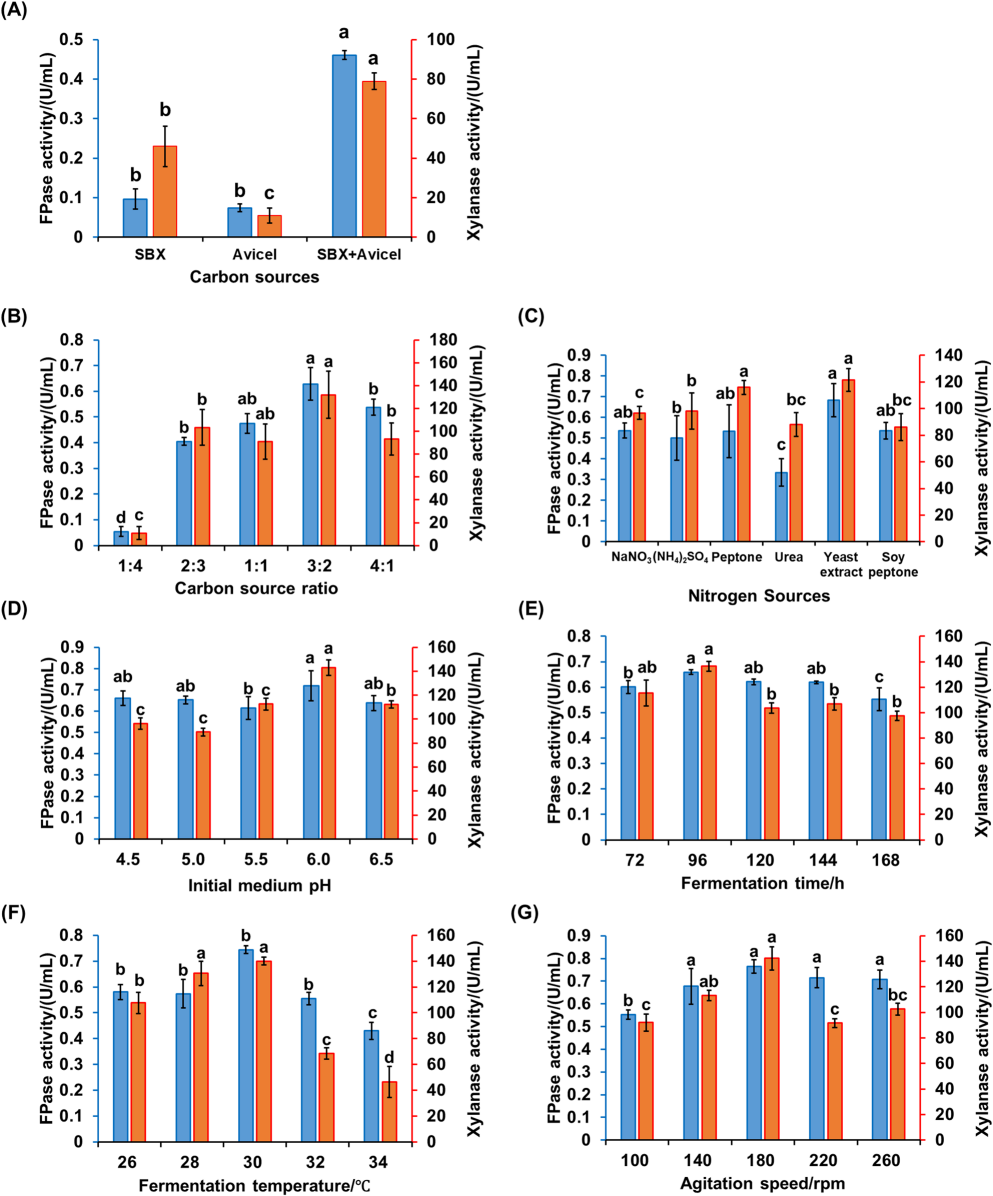
Systematic and sequential optimization of cellulase and xylanase co-production by *P. oxalicum* strain UNN1 using a mixed carbon source. **A** Effects of single or mixed carbon sources (total 10 g/L) on enzyme activities. **B** Effect of sugarcane bagasse xylan (SBX): Avicel mass ratios (total 10 g/L) on FPase and xylanase activities. **C** Effect of types of nitrogen sources (4 g/L) on FPase and xylanase activities. **D** Effect of the initial medium pH on FPase and xylanase activities. **E** Effect of fermentation time (h) on FPase and xylanase activities. **F** Effect of fermentation temperature (°C) on FPase and xylanase activities. **G** Effect of agitation speed (rpm) on FPase and xylanase activities. Data are presented as mean ± SD (*n* = 3), bars marked with different letters indicate significant differences (*P* < 0.05). FPase: filter paper cellulase

In addition, to further evaluate the cellulolytic capacity of strain UNN1, Avicel was supplemented to the SmF medium, including SBX. When a 1:1 (w/w; 10 g/L) mixture of SBX and Avicel was used as the carbon source, the FPase and xylanase activities reached (0.46 ± 0.06) U/mL and (78.93 ± 1.41) U/mL, respectively. These values were significantly higher than those obtained with either SBX or Avicel alone as the carbon source (Fig. 3A). Mixed carbon sources provide a more diverse range of substrates, leading to more effective enzyme induction and significantly increasing enzyme production. For instance, when crystalline cellulose was mixed with mandarin pomace, it synergistically boosted the production of cellulases and xylanases by basidiomycetes (Metreveli et al. 2021).

Subsequently, a sequential single-factor optimization of enzyme-production conditions was conducted, covering the SBX: Avicel ratio (10 g/L), types of nitrogen sources (4 g/L), initial medium pH, fermentation time, fermentation temperature, and agitation speed. The optimal parameters were determined as follows: a SBX: Avicel ratio of 3:2 (w/w), which resulted in the highest activities of FPase and xylanase, reaching (0.63 ± 0.06) U/mL and (131.87 ± 20.70) U/mL, respectively (Fig. 3B); yeast extract at 4.0 g/L, yielding FPase and xylanase activities of (0.68 ± 0.08) U/mL and (121.54 ± 8.45) U/mL, respectively (Fig. 3C); an initial medium pH of 6.0, resulting in FPase and xylanase activities of (0.72 ± 0.07) U/mL and (143.03 ± 6.60) U/mL, respectively (Fig. 3D); a fermentation time of 96 h, with FPase and xylanase activities of (0.66 ± 0.01) U/mL and (136.42 ± 3.78) U/mL, respectively (Fig. 3E); a fermentation temperature of 30 °C, yielding FPase and xylanase activities of (0.74 ± 0.02) U/mL and (140.00 ± 3.09) U/mL, respectively (Fig. 3F)。and agitation at 180 rpm, resulting in FPase and xylanase activities of (0.76 ± 0.03) U/mL and (142.32 ± 9.19) U/mL, respectively (Fig. 3G). These optimized conditions significantly enhanced the production of both cellulase and xylanase, but the xylanase activity did not reach the levels achieved with 25 g/L SBX as the carbon source, where it reached (191.22 ± 4.27) U/mL. The likely reason is that we limited the maximum addition of mixed carbon sources to 10 g/L, which did not achieve the optimal inducing substrate concentration. Producing high-activity enzymes with less raw material is the ideal scenario for industrial applications. Interestingly, in this study, peak enzyme production was achieved within 96 h using the optimized mixed carbon source (SBX: Avicel). In contrast, even with ball-milled corn stover as an inducer, peak cellulase production by *T. reesei* RutC-30 typically requires over 120 h (He et al. 2024). This reduction in fermentation duration could contribute to lower energy and time costs in industrial operations.

Moreover, the reason that urea is no longer the optimal nitrogen source for fermentation is likely due to the activation of different metabolic pathways in microorganisms caused by changes in the types of carbon sources and the C/N ratio, which in turn affects the selection and utilization of nitrogen sources (Wang et al. 2023). For instance, Wang et al. (2023) found that under different C/N ratios, various substrates significantly influence the biological transformation mechanisms of NO_3_^−^ and SO_4_^2−^, as well as the potential carbon metabolic pathways (Wang et al. 2023). Furthermore, mixed carbon sources advanced the fermentation time to achieve maximum enzyme production, which is more advantageous for industrial applications by saving costs and energy consumption. Finally, there were slight changes in the optimal environmental factors such as fermentation temperature, agitation speed, and initial medium pH, indicating that the combination of these parameters effectively promotes the enzymatic activities.

While the single-factor design was adopted in this exploratory stage due to its simplicity and interpretability, especially when dealing with multiple variables, it does carry the limitation of being unable to detect key interactions between parameters—such as those between carbon concentration, C/N ratio, and pH. In future work, to address this and further refine the fermentation process, we intend to scale up using a statistical design of experiments (e.g., Plackett–Burman followed by RSM), while continuing to explore the broader application of these enzymes in biotechnological processes.

### Effects of pH and temperature on cellulase and xylanase activity and stability

**Fig. 4:**
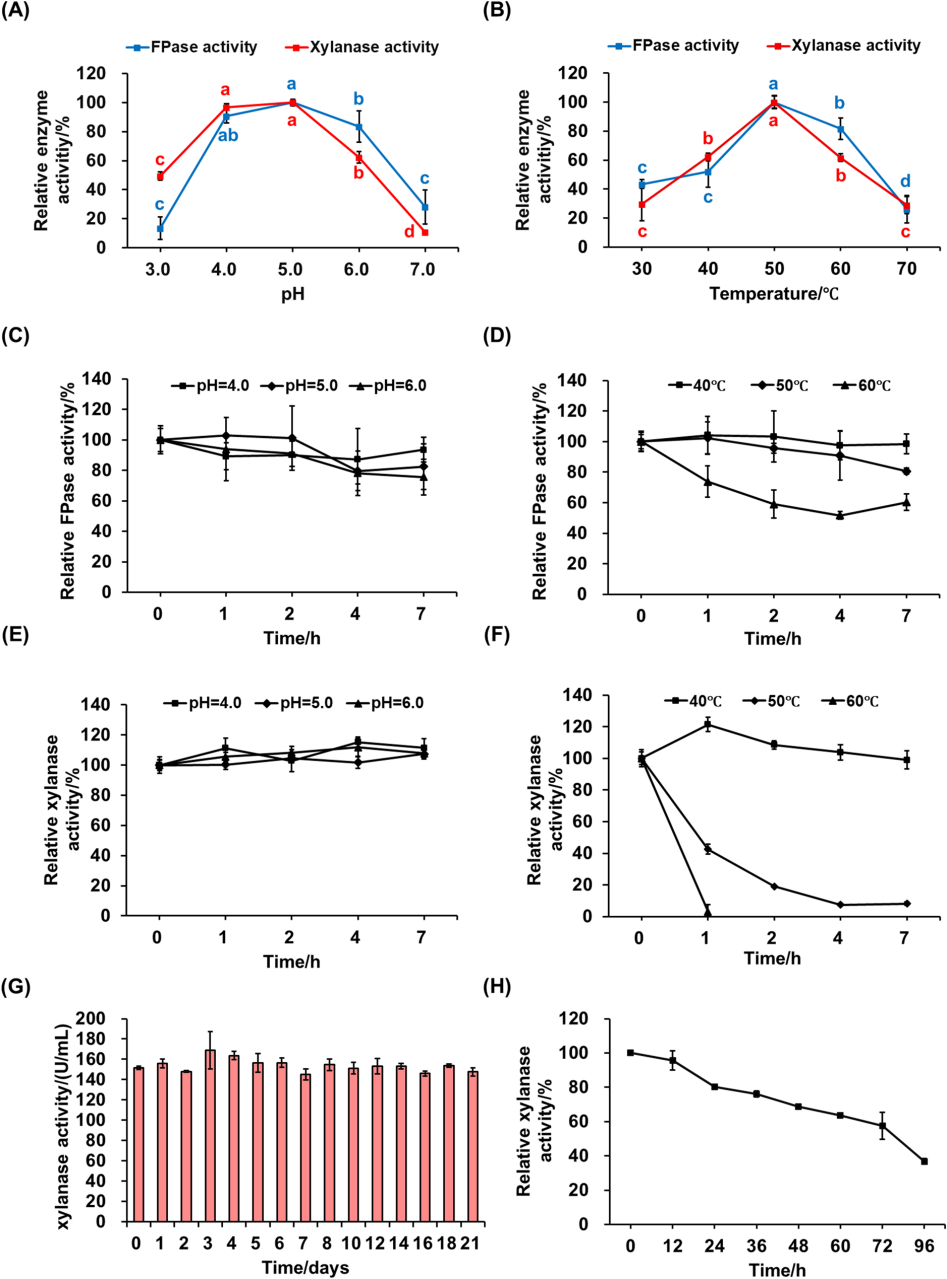
The effect of pH (**A**, **C**, **E**) and temperature (**B**, **D**, **F**, **G**, **H**) on the activity and stability of cellulase and xylanase secreted by *P. oxalicum* strain UNN1. **A** Determination of the optimum reaction pH. (**B**) Determination of the optimum reaction temperature. **C** Low-temperature pH stability determination of cellulase at 4 °C and pH 4.0–6.0. **D** Thermal stability determination of cellulase at 40–60 °C and pH 5.0. **E** Low-temperature pH stability determination of xylanase at 4 °C and pH 4.0–6.0. **F** Thermal stability determination of xylanase at 40–60 °C and pH 5.0. **G** Assessment of the long-term storage stability of xylanase in the crude enzyme mixture at 4 °C for 21 days. **H** Stability of crude xylanase at 40 °C. FPase: filter paper cellulase

The optimal pH analysis for cellulase and xylanase produced by strain UNN1 under SmF conditions revealed that both enzymes exhibited peak activity at pH 5.0 when tested at 50 °C. Additionally, the enzymes retained over 80% of their maximum FPase activity and 60% of their maximum xylanase activity within the pH range of 4.0 to 6.0. However, the FPase activity declined sharply to below 20% at pH 3.0, while it maintained 28.17% of its maximum activity at pH 7.0. Conversely, the xylanase activity plummeted to below 20% at pH 7.0, yet it still preserved 49.49% of its maximum activity at pH 3.0 (Fig. 4A). These findings suggest that the cellulase and xylanase are both acidic enzymes, capable of maintaining high activity within the industrially relevant pH range of 4.0–6.0. Notably, cellulase is more sensitive to acidic shifts, whereas xylanase shows greater sensitivity to alkaline deviations.

The thermal profiles of cellulase and xylanase highlight their mesophilic nature, with optimal activity at 50 °C under acidic conditions (pH 5.0). Furthermore, the enzymes retained more than 50% of their maximum FPase activity and over 60% of their maximum xylanase activity within the 40 °C to 60 °C range. Notably, the FPase preserved 81.70% of its peak activity at 60 °C. This robustness is notable, yet a marked decline in activity is observed when temperatures rise to 70 °C, with FPase and xylanase activities plummeting to 26.17% and 28.87%, respectively (Fig. 4B). This decline underscores the enzymes’ sensitivity to elevated temperatures, indicating that industrial processes should avoid prolonged exposure to temperatures above 70 °C to preserve enzyme functionality. This temperature sensitivity suggests that precise temperature control is necessary in the design of industrial processes to ensure that the enzymes maintain their catalytic efficiency throughout the production process, thereby improving productivity and cost-effectiveness.

Subsequent assessments of low-temperature pH stability revealed that both cellulase and xylanase exhibited remarkable acid stability (Fig. 4C, E, G). FPase demonstrated relative stability at 4 °C across a pH range of 4.0–6.0, retaining over 75% of its initial activity after 7 h of incubation (*p* < 0.05; Fig. 4C). Interestingly, xylanase maintained nearly 100% of its initial activity after 7 h at 4 °C within the same pH range (Fig. 4E). Extending the low-temperature storage period further highlighted the robustness of the crude enzyme mixture, with xylanase activity remaining virtually unchanged after 21 days of storage at 4 °C and the optimal pH of 5.0 (Fig. 4G). This highlights the excellent low-temperature storage stability of xylanase, which extends shelf life, reduces costs, and maintains efficiency, benefiting long-term storage and transport in industrial applications.

Regarding thermal stability, cellulase exhibited no significant change in activity after being treated at 40 °C for 7 h or at 50 °C for 4 h, and still exhibited over 80% and 50% of its residual activity after 7 h at temperatures between 50 °C and 60 °C (*p* < 0.05). In contrast, xylanase remained fully active at 40 °C (2–7 h), with a significant increase of 21.48% in activity after 1 h of incubation (*p* < 0.05). However, its half-life at 50 °C was less than 1 h, with only 42.60% of residual activity remaining, and the enzyme was almost completely inactivated after just 1 h at 60 °C (Fig. 4D, F). These results indicate that cellulase possesses markedly higher thermostability than the xylanase. Therefore, the crude enzyme mixture is more suitable for industrial applications focused primarily on the hydrolysis of cellulose-based substrates, especially in processes requiring higher temperatures. The significant increase in xylanase activity after 1 h of incubation at 40 °C (+ 21.48%) suggests that the enzyme may undergo beneficial conformational changes or enhanced substrate binding, a phenomenon consistent with thermal activation reported in other industrial enzymes (Nam 2024). This observed activation, coupled with the enzyme’s exceptionally low-temperature storage stability—retaining nearly full activity after 21 days at 4 °C and pH 5.0—highlights the robust and industrially favorable nature of the UNN1 xylanase. When compared with recent studies on fungal xylanases (e.g., Zhao et al. 2024; which reviewed regulatory mechanisms in *Penicillium* and *Trichoderma*), the performance of UNN1 in terms of both yield (191.22 U/mL using SBX) and stability under storage conditions demonstrates clear practical advantages. Further mechanistic studies, such as detailed kinetic analyses and structural investigations, are warranted to fully elucidate the activation pathway. Moreover, the weak stability of xylanase activity at its optimal reaction temperature of 50 °C somewhat restricts the direct application of the crude enzyme from strain UNN1 in the industrial degradation of lignocellulose. Further research is needed to explore how to enhance its stability at high temperatures through genetic or protein engineering.

In addition, we referred to the hydrolysis temperature from our laboratory’s previous work (Metreveli et al. 2021) and the stability temperature of the crude xylanase determined in Fig. 4F, and decided to further investigate the half-life of the crude xylanase at 40 °C to define the range of hydrolysis time for enzymatic hydrolysis experiments. As shown in Fig. 4H, the relative activity of xylanase was 68.82% at 48 h, decreased to 57.42% at 72 h, and further dropped to 36.82% at 96 h. These results indicate that the crude xylanase exhibits high stability at 40 °C, maintaining catalytic activity over an extended period.

### Effect of various metal ions on cellulase and xylanase activity

**Fig. 5:**
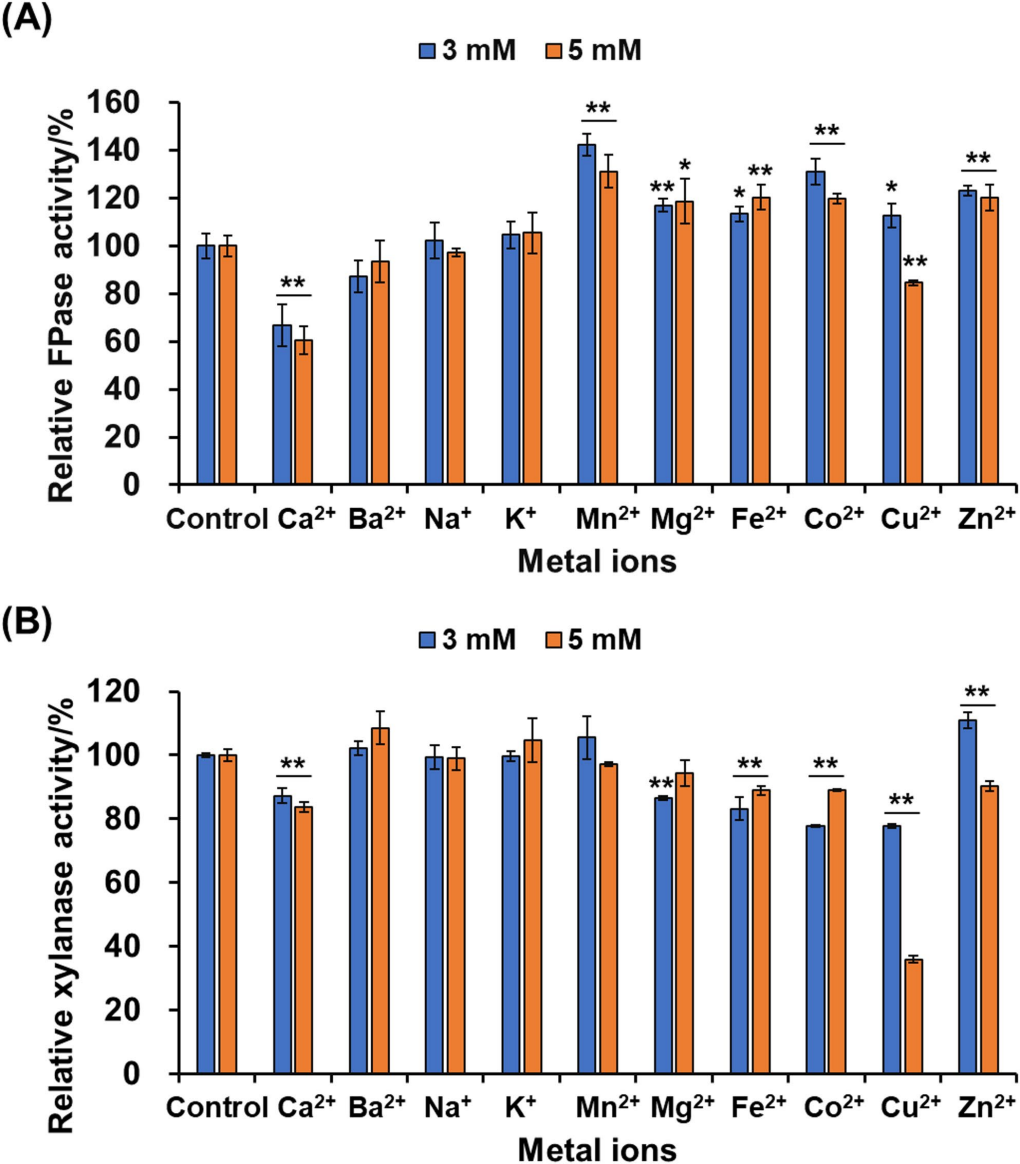
Effect of metal ions on FPase (**A**) and xylanase (**B**) activity. FPase: filter paper cellulase

To further investigate the influence of industrially relevant metal ions on cellulase and xylanase activities, the crude enzyme mixture was diluted with metal-ion-containing buffers (pH 5.0) at final concentrations of 3 mM and 5 mM and incubated at room temperature (~ 25 °C) for 30 min before residual activity was determined. The results revealed that Ca²⁺ at both concentrations markedly suppressed the activities of FPase and xylanase, reducing them by 33.25–39.47% and 12.78–16.35%, respectively (Fig. 5A, B). The high concentration of Ca²⁺ (in this study, the final concentrations after additional supplementation of 3 mM and 5 mM both exceeded 8 mM) may competitively bind to the -SH groups (cysteine residues) in the enzyme’s active site, inducing conformational changes. Furthermore, charge shielding effects may weaken electrostatic and hydrogen bond interactions between the enzyme and its substrate, further inhibiting catalytic efficiency, ultimately leading to reduced cellulase and xylanase activity (Yang et al. 2019). In contrast, Mn²⁺ significantly enhanced FPase activity by 31.19–42.33%, whereas xylanase activity remained unaffected (Fig. 5A, B). This result is consistent with the finding that manganese peroxidase (MnP) from *Phanerochaete chrysosporium* can enhance cellulase activity (Min et al. 2022). Chen et al. (2018) revealed that Mn²⁺ stimulates cellulase production and demonstrated that it upregulates cellulase gene expression through calcium channels and calcium signaling.

Interestingly, Mg²⁺, Fe²⁺, Co²⁺, Cu²⁺, and Zn²⁺ at both concentrations significantly stimulated FPase activity (12.69–30.98% increase; *p* < 0.05), except for 5 mM Cu²⁺, which exerted a notable inhibitory effect (15.39% decrease; *p* < 0.05) (Fig. 5A). Conversely, these five ions generally inhibited xylanase activity (9.75–64.01% decrease; *p* < 0.05), with the exceptions of 3 mM Zn²⁺, which slightly enhanced the activity by 10.97% (*p* < 0.05), and 5 mM Mg²⁺, which had no effect (Fig. 5B). The differential effects of metal ions on FPase and xylanase activities may be attributed to their distinct active site architectures, substrate specificities, and catalytic mechanisms, where these ions differentially modulate enzyme conformation or substrate interaction (Hou et al. 2023). Therefore, the varying coordination preferences of metal ions can stabilize FPase while disrupting xylanase structure or catalysis. Finally, Ba²⁺, Na⁺, and K⁺ showed no significant influence on either enzyme. It is noteworthy that 5 mM Cu²⁺ strongly suppressed both cellulase and xylanase activities (Fig. 5A, B). The mechanism by which 5 mM Cu²⁺ inhibits glycoside hydrolases is not yet well understood. However, due to the toxicity of copper, studies have shown that adding 10 µM CuSO₄ is sufficient to kill *P. oxalicum* strains (Lin et al. 2021). Additionally, 5 mM Cu²⁺ inhibits the growth, mycotoxin production, and hydrolytic enzymes of *Fusarium incarnatum* (Al-Rajhi et al. 2022). It has also been reported that 10 mM Cu²⁺ causes a significant reduction of over 60% in the activity of xylanase Xyl *PP* (De Camargo et al. 2022). We have previously reported that 5 mM Cu²⁺ exhibits a highly significant inhibitory effect on the activity of xylanase BsXynA (Zhang et al. 2025), which aligns with the findings of this study. These results emphasize the need to prevent contamination with Ca²⁺ and Cu²⁺ during industrial processes.

Preliminary exploration of the application of cellulase and xylanase produced by *P. oxalicum* strain UNN1 in the degradation of agricultural residues.

To evaluate the hydrolysis efficiency of crude cellulase and xylanase produced by strain UNN1 on various agricultural residues—including peanut shells, corn cobs, orange peels, pomelo peels, rice straws, wheat bran, and sugarcane bagasse—a hydrolysis reaction was carried out in a 50 mL system containing 10 g/L of each substrate. The reaction was initiated by the addition of 10 mL of crude enzyme solution, which provided approximately 7 U of FPase and 1,400 U of xylanase activity, resulting in a final enzyme dosage of 14 U FPase and 2,800 U xylanase per gram of dry biomass. Hydrolysis was performed at 40 °C with agitation at 180 rpm for 48 h, after which the yield of reducing sugars was determined.

**Fig. 6:**
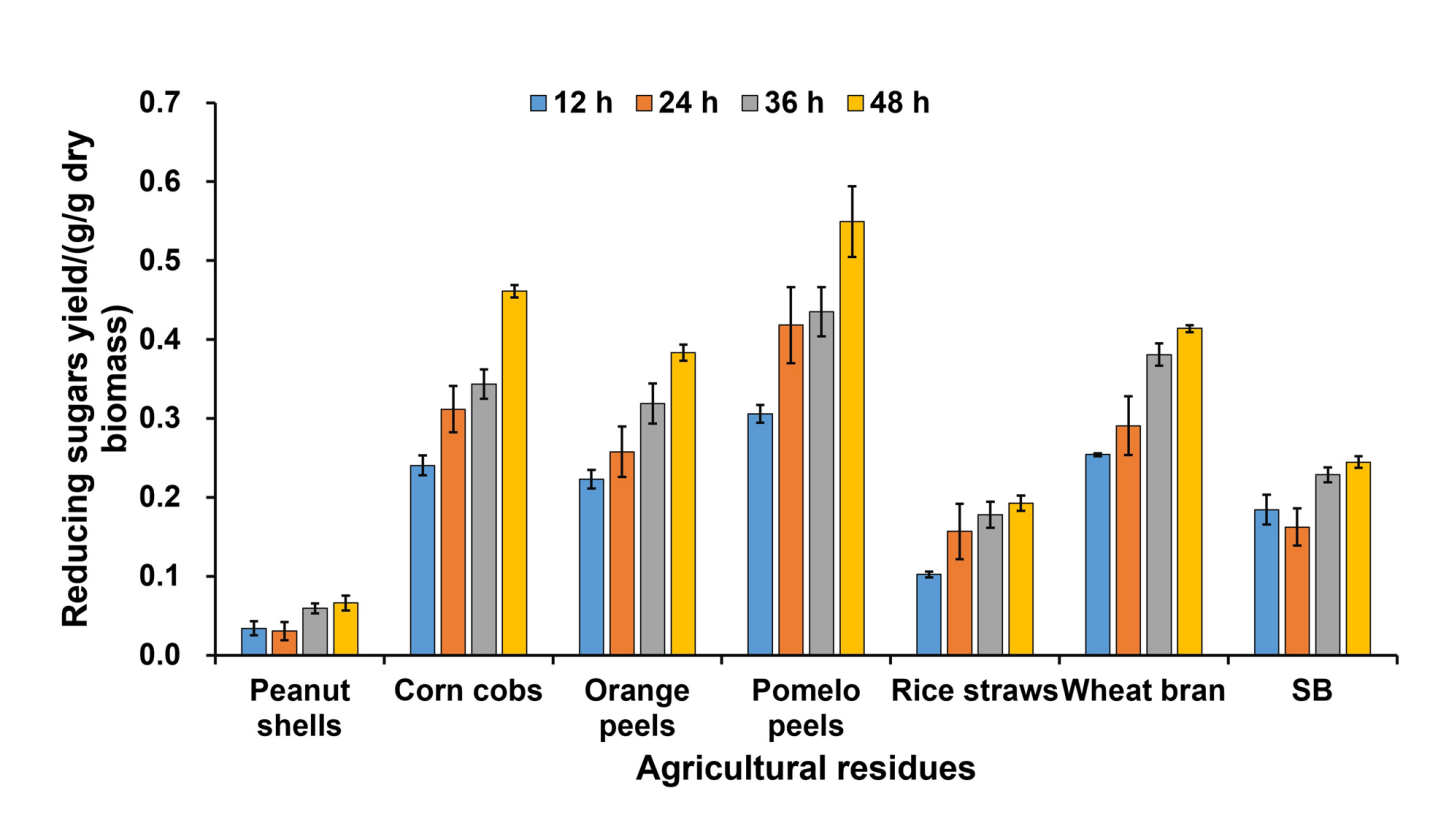
Reducing sugar yield in different non-alkaline-extracted agricultural residues during enzymatic hydrolysis for 48 h. SB: sugarcane bagasse

As shown in Fig. 6, the reducing sugar yield of all substrates increased with hydrolysis time, reflecting their distinct lignocellulosic compositions (Table 3). Higher yields were obtained from pomelo peels, corn cob, wheat bran, and orange peels, with yields of 0.549, 0.461, 0.414, and 0.383 g/g dry biomass, respectively. These substrates are characterized by relatively low lignin contents (Table 3, 30.24% for pomelo peel, 11.9–15% for corn cob, 16–21% for wheat bran, and 3–6.93% for orange peel) and structurally accessible matrices, which facilitated efficient enzymatic degradation under the given enzyme loading (Zhang et al. 2024; Sunesh et al. 2025; Ghaffar et al. 2013; Junker et al. 2024).

**Table 3:** Typical composition ranges of the agricultural residues used in this study

Substrate	Cellulose (%)	Hemicellulose (Xylan) (%)	Lignin (%)	References
Peanut shells	45	6	36	Sawargaonkar et al. 2024
Corn cobs	31-38.8	44.4–50.5	11.9–15	Sunesh et al. 2025; Zielińska et al. 2024
Orange peels	14.4–21.1	10.9–13.8	3-6.93	Junker et al. 2024; Pélagie et al. 2022; Pocan et al. 2018
Pomelo peels	46.22	18.84	10.24	Zhang et al. 2024
Rice straws	24–47	19-32.1	5–24	Ghaffar et al. 2013; Sunesh et al. 2025; Zielińska et al. 2024
Wheat bran	22–40	20–38	16–21	Ghaffar et al. 2013; Sunesh et al. 2025; Zielińska et al. 2024
SB (sugarcane bagasse)	25–45	27–32	18–28	Ling et al. 2022; Moreira et al. 2023; Sunesh et al. 2025; Zielińska et al. 2024

Moderate hydrolysis efficiency was observed for sugarcane bagasse (SB, 0.245 g/g dry biomass) and rice straw (0.192 g/g dry biomass). Although SB has a lignin content (Table 3, 18–28%) comparable to that of wheat bran, its higher cellulose crystallinity and more complex lignin-carbohydrate associations likely contributed to its greater recalcitrance under the same enzyme conditions (Ling et al. 2022; Moreira et al. 2023). For rice straw, despite its variable lignin content (Table 3, 5–24%), the limited hydrolysis may be attributed not only to its compact structural organization but also to the relatively high cellulose content (Table 3, 24–47%) which, under the current cellulase dosage, may have been insufficient for complete degradation (Ghaffar et al. 2013; Sunesh et al. 2025). Similarly, the moderate yield from SB may also reflect an imbalance between its substantial cellulose fraction (Table 3, 25–45%) and the available cellulase activity.

In contrast, peanut shells exhibited the lowest sugar yield (Table 3, 0.066 g/g dry biomass), consistent with their high lignin content (Table 3, 36%) and rigid, densely lignified structure, which severely restricted enzyme accessibility and hydrolysis efficiency (Sawargaonkar et al. 2024). These results indicate that the crude enzymes from strain UNN1 are particularly effective for the degradation of agricultural residues with low to moderate lignin content and an open fibrous structure, such as pomelo peels, corn cobs, wheat bran, orange peels, and rice straw—especially those with higher cellulose content—further optimization of enzymatic hydrolysis conditions, including increased cellulase supplementation, adjusted enzyme formulations, or the application of physicochemical pretreatments, may be necessary to enhance sugar release and process efficiency.

To gain deeper insight into the kinetic nature of the differences in hydrolysis efficiency among substrates, we performed nonlinear regression analysis (one-phase association model) on the reducing sugar accumulation curves over 48 h. As shown in Table 4, the model provided a good fit for corn cobs, orange peels, pomelo peels, rice straws, and wheat bran (R² > 0.72), indicating that their hydrolysis generally followed first-order kinetics. However, poorer fits were obtained for peanut shells (R² = 0.55) and sugarcane bagasse (R² = 0.25), suggesting that the hydrolysis of these lignin-rich substrates might be influenced by complex factors (e.g., substrate inhibition, enzyme inactivation), deviating from the simple model. The fitted parameters revealed that pomelo peels had the highest theoretical saccharification potential (Plateau = 0.538 g/g dry biomass), while peanut shells had the lowest (0.096 g/g). Sugarcane bagasse exhibited the fastest apparent initial rate (K = 0.103 h⁻¹) but the shortest half-time (6.7 h), implying a rapid initial hydrolysis followed by a sharp slowdown due to structural barriers. In contrast, peanut shells showed the slowest rate (K = 0.024 h⁻¹) and the longest half-time (29 h). This analysis demonstrates that for structurally loose feedstocks, hydrolysis is smooth and predictable, whereas for highly recalcitrant feedstocks, hydrolysis exhibits complex, non-linear kinetics, necessitating pretreatment to disrupt structural barriers for efficient saccharification.

**Table 4:** Kinetic parameters derived from one-phase association model fitting for the enzymatic hydrolysis of different agricultural residues over 48 h

Substrate	Plateau (g/g dry biomass)	K (h⁻¹)	Half-time (h)	*R*²	95% CI of Plateau (g/g dry biomass)
Peanut shells	0.0962	0.0239	29.06	0.548	0.0518–???
Corn cobs	0.4703	0.0488	14.21	0.776	0.3875–0.6741
Orange peels	0.3842	0.0575	12.05	0.727	0.3242–0.5111
Pomelo peels	0.5383	0.0634	10.94	0.733	0.4621–0.6830
Rice straws	0.2040	0.0590	11.75	0.744	0.1722–0.2683
Wheat bran	0.4313	0.0578	11.99	0.767	0.3716–0.5485
Sugarcane bagasse	0.2273	0.1032	6.718	0.247	0.1912–0.2934

Note: Parameters were obtained by fitting the time-course reducing sugar yield data to a one-phase association model: *Y* = *Y*_0_ + (*Plateau* – *Y*_0_)*(1 – *e*^− k*t^), with *Y*_0_ constrained to 0. CI: confidence interval. “???” indicates that the upper limit could not be reliably estimated from the data

**Table 5:** Analysis of variance (ANOVA) for the regression model in optimizing the reducing sugar yield

Source	Sum of squares	df	Mean square	F-value	*p*-value	Significance
Model	0.9893	9	0.1099	10.53	0.0026	significant
A- Xylanase dosage	4.500E-06	1	4.500E-06	0.0004	0.9840	
B- pH of hydrolysate	0.0151	1	0.0151	1.45	0.2676	
C- Hydrolysis temperature	0.0084	1	0.0084	0.8094	0.3982	
AB	0.0066	1	0.0066	0.6285	0.4539	
AC	0.0005	1	0.0005	0.0464	0.8357	
BC	0.0428	1	0.0428	4.10	0.0824	
A²	0.3750	1	0.3750	35.93	0.0005	**
B²	0.4084	1	0.4084	39.12	0.0004	**
C²	0.0518	1	0.0518	4.96	0.0611	
Residual	0.0731	7	0.0104			
Lack of fit	0.0549	3	0.0183	4.03	0.1058	not significant
Pure error	0.0182	4	0.0045			
Cor total	1.06	16				
R^2^=0.9312 Adj R^2^ = 0.8428						

Note: *p* < 0.01 indicates highly significant, marked with **; *p* < 0.05 indicates significant, marked with *

### Preliminary exploration of the application of *P. oxalicum* strain UNN1 in hydrolyzing SBX to produce reducing sugars

In addition, to explore the ability of xylanase secreted by strain UNN1 to hydrolyze SBX and produce reducing sugars or weakly reducing prebiotic xylooligosaccharides, the hydrolysis medium containing 10 g/L SBX was subjected to hydrolysis for a specified duration, after which the yield of reducing sugars was measured. Subsequently, xylanase dosage (240 U/g, 480 U/g, and 720 U/g SBX; factor A), pH of hydrolysate (5.0, 5.5, 6.0; factor B), and hydrolysis temperature (30 °C, 40 °C, 50 °C; factor C) were selected for the single-factor optimization of hydrolysis conditions (with a hydrolysis time range of 0–48 h) and response surface optimization (with a hydrolysis time of 12 h) (Table 1).

**Fig. 7:**
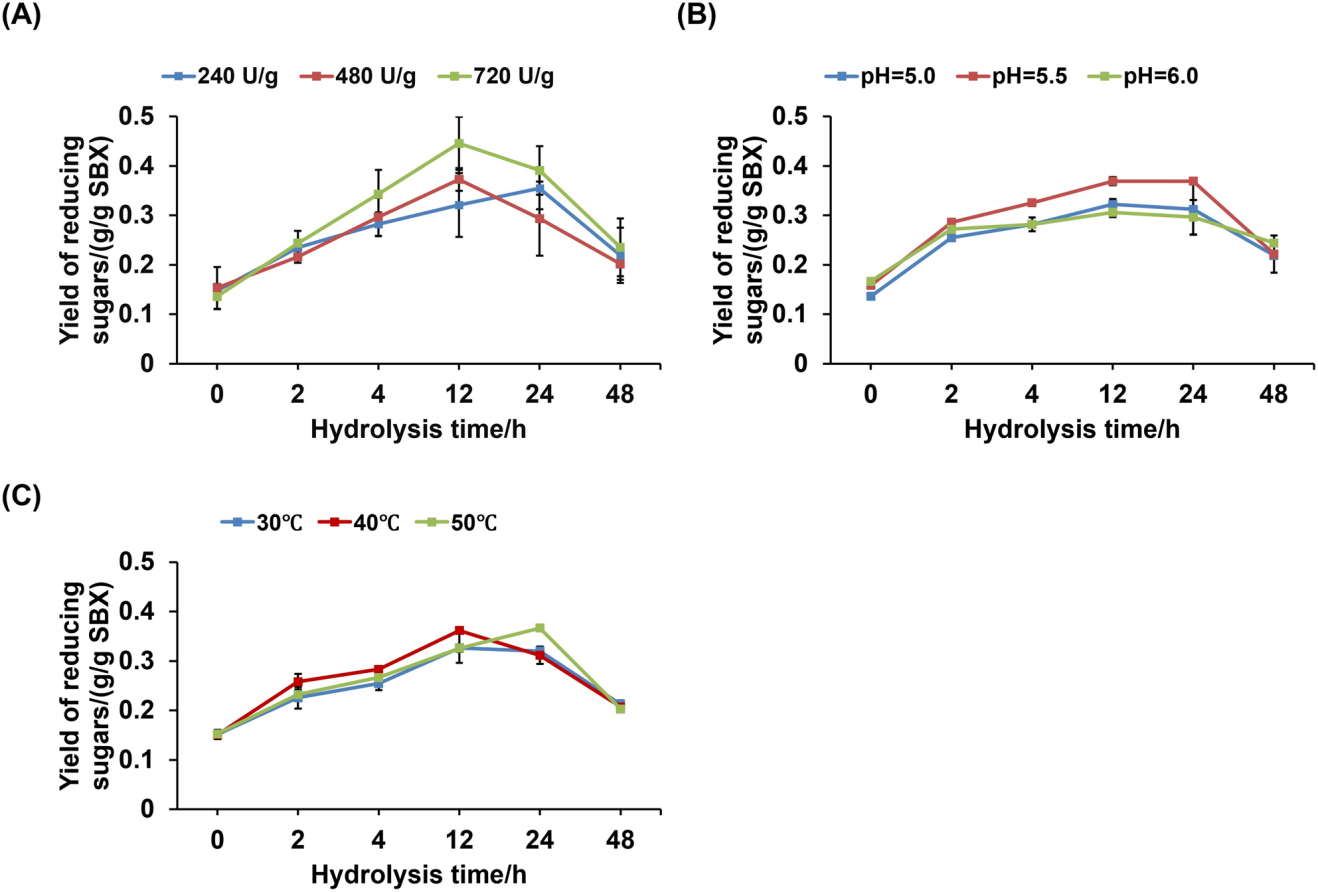
Effects of different conditions on the hydrolysis of sugarcane bagasse xylan. **A** Effect of xylanase dosage on reducing sugar yield at different hydrolysis times; **B** Effect of hydrolysate pH on reducing sugar yield at different hydrolysis times; **C** Effect of hydrolysis temperature on reducing sugar yield at different hydrolysis times. SBX: sugarcane bagasse xylan

The results of the single-factor experiments are shown in Fig. 7A-C. The yield of reducing sugars reached a maximum of (0.446 ± 0.054) g/g SBX when hydrolysis was conducted with 720 U/g SBX xylanase for 12 h. When the xylanase dosage was 480 U/g SBX and the hydrolysis time was 12 h, or when the xylanase dosage was 240 U/g SBX and the hydrolysis time was 24 h, the concentration of reducing sugars reached the highest levels of (0.373 ± 0.023) g/g and (0.355 ± 0.042) g/g SBX, respectively (Fig. 7A). The possible reason for these results is that an increased enzyme dosage enhances the binding frequency between the enzyme and the substrate, allowing more xylan molecules to be acted upon by the enzyme within the same time frame, thus accelerating the hydrolysis reaction rate and producing more reducing sugars (Lee et al. 2024). The delayed peak of reducing sugar accumulation at the dosage of 240 U/g SBX also confirms this speculation. Figure 7B and C illustrate the effects of hydrolysate pH and hydrolysis temperature on the enzymatic hydrolysis of SBX, both exhibiting almost identical trends. As the hydrolysis time increased, the yield of reducing sugars showed an increasing trend, and all reached a peak at 12–24 h with 0.306 g/g to 0.369 g/g SBX. Based on these observations, the yield of reducing sugars after 12 h of hydrolysis was used as the response value, and a response surface methodology (RSM) based on the Box-Behnken design (BBD) was employed to optimize the experimental conditions. Data were analyzed using Design-Expert 13.0 (Stat-Ease, Inc., Minneapolis, MN, USA) to establish a quadratic regression model. As shown in Table 5, the model was highly significant (*p* < 0.01), with a coefficient of determination (R²) of 0.9312, indicating a strong correlation between observed and predicted values. The lack-of-fit test was not significant (*p* = 0.1058 > 0.05), suggesting that the model fits the experimental data well with minimal unexplained variation. The F-value serves as an indicator of the relative significance of model terms, with higher values reflecting greater influence on the response. In this model, linear terms were not statistically significant (*p* > 0.05), whereas quadratic terms exhibited significant effects (*p* < 0.05), implying a nonlinear relationship between the independent variables and the response. The adjusted R² value of 0.8428 and a low coefficient of variation (CV = 3.07%) further confirm the model’s reliability, reproducibility, and predictive capability. A second-order polynomial equation was generated with reducing sugar yield as the response variable: *Y* = 3.66 + 0.0007 A + 0.0435B + 0.0325 C + 0.0405AB-0.011AC-0.1035BC-0.2984A^2^-0.3115B^2^-0.111C^2^. This equation adequately describes the influence of the three factors on reducing sugar yield and can be used for process analysis and optimization.

**Fig. 8:**
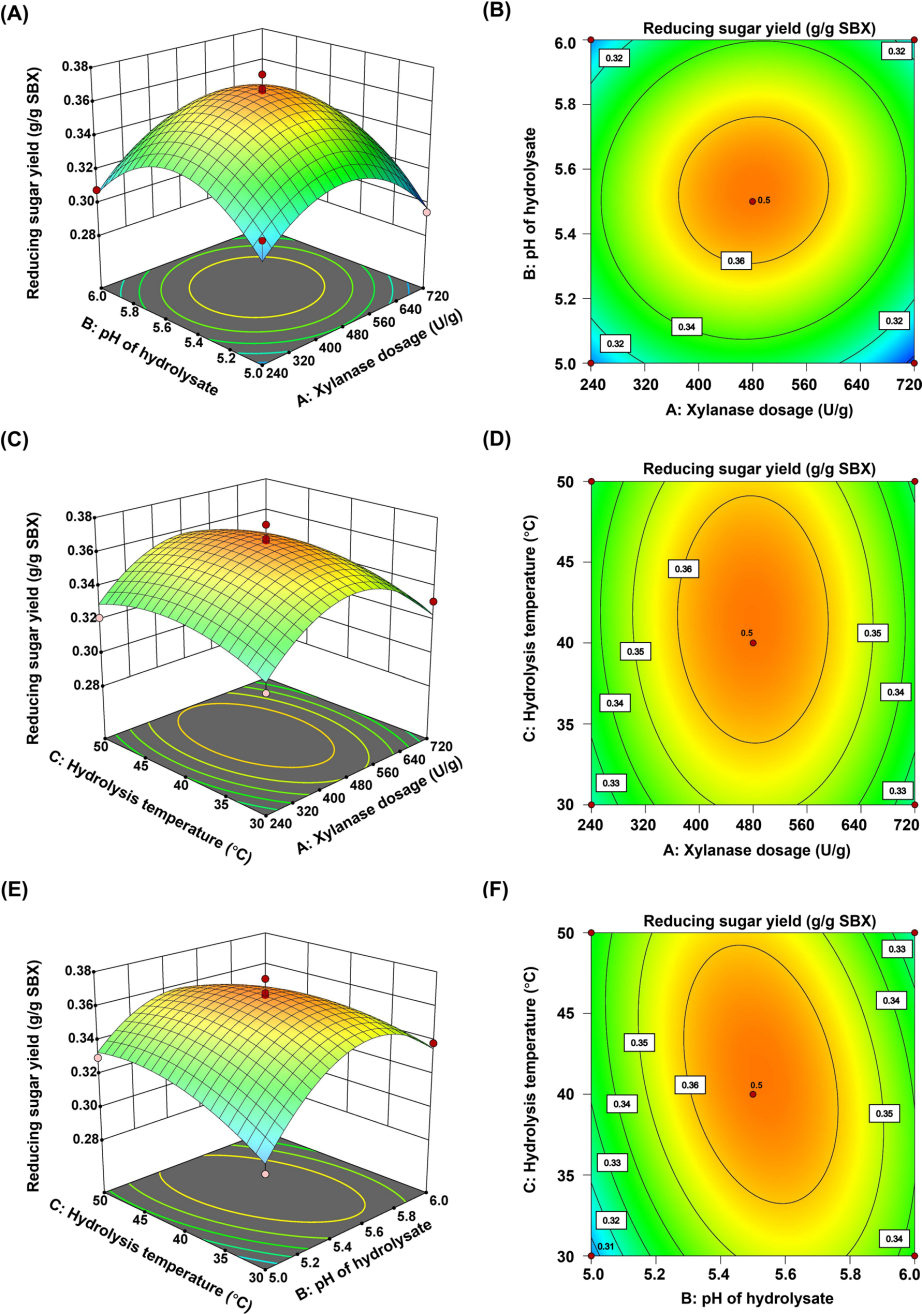
Three-dimensional response surface plots and contour plots of the effects of xylanase dosage (**A**), pH of hydrolysate (**B**), and hydrolysis temperature (**C**) on the yield of reducing sugars. SBX: sugarcane bagasse xylan

The interactive effects of xylanase dosage (A), pH of hydrolysate (B), and hydrolysis temperature (C) on the yield of reducing sugars are shown in Fig. 8A-F. In the 3D surface plots, the steeper the surface in the direction parallel to the coordinate axis, the greater the influence of the corresponding factor on the response value. In the contour plots, the direction of the long axis of the ellipse reveals the dominant direction of the interaction. The smaller the spacing between different contour lines, the greater the influence of the factor on the response value. Typically, the flatter and more densely packed the contour plot, the more significant the interaction between the two variables.

The interaction between factors A and B is shown in Fig. 8A and B. The 3D surface plot indicates that the response value, the yield of reducing sugars, changes steeply in both factor directions, suggesting that both A (xylanase dosage) and B (pH of hydrolysate) have a significant impact on the yield of reducing sugars. The nearly circular shape of the contour plot indicates that the interaction between the two factors is not significant.

The interaction between factors A and C is shown in Fig. 8C and D. The 3D surface plot shows that the response value changes steeply in the direction of factor C (hydrolysis temperature) and more gently in the direction of factor A (xylanase dosage), indicating that hydrolysis temperature (C) has a greater impact on the yield of reducing sugars. The elliptical shape of the contour plot, with the long axis in the direction of factor C, indicates a significant interaction between the two factors, with hydrolysis temperature being the primary influence.

The interaction between factors B and C is shown in Fig. 8E and F. The 3D surface plot shows that the response value changes steeply in the direction of factor C (hydrolysis temperature) and more gently in the direction of factor B (hydrolysate pH), indicating that hydrolysis temperature (C) has a greater impact on the yield of reducing sugars. The elliptical shape of the contour plot, with the long axis in the direction of factor C, indicates a significant interaction between the two factors, with hydrolysis temperature being the primary influence.

Based on the regression model and aimed at maximizing the reducing sugar yield, the predicted optimal conditions were determined as enzyme loading of 480.60 U/g SBX, hydrolysis temperature of 41.24 °C, and pH 5.52, yielding a predicted output of 0.367 g/g SBX. For practical and operational feasibility, the conditions were slightly adjusted to an enzyme dosage of 480 U/g SBX, temperature of 40 °C, and pH 5.5. Under these modified conditions, the experimental maximum reducing sugar yield reached (0.355 ± 0.011) g/g SBX, which deviates from the predicted value by less than 5.0%, confirming the model’s accuracy and reliability.

A preliminary application test demonstrated that the crude xylanase from strain UNN1 effectively hydrolyzes SBX to produce reducing sugars. This result suggests that strain UNN1 has strong potential for direct utilization in the enzymatic conversion of xylan-rich agricultural residues—such as sugarcane bagasse and corn cobs—into fermentable sugars, paving the way for its application in sustainable biorefineries and high-value bioproduct development.

Since only total reducing sugars were measured in this study, the monosaccharide and oligosaccharide composition of the hydrolysate remains unknown. In future work, advanced techniques such as ultra-performance liquid chromatography (UPLC) or high‑performance anion‑exchange chromatography with pulsed amperometric detection (HPAEC‑PAD) should be employed to precisely quantify individual sugars (e.g., xylose, glucose) and oligosaccharide fractions. This will enable a complete characterization of the hydrolysis products and support downstream applications such as microbial fermentation or high-value biochemical production.

### Integrated economic potential

Building upon the above research outcomes, the technical system established in this study demonstrates significant economic potential. Combined with the high enzyme activity yield (191.22 U/mL) achieved and the excellent storage stability (no significant activity loss over 21 days), the overall costs associated with the production, storage, and transportation of this enzyme preparation are expected to be substantially lower than those of traditional fermentation processes. Although precise economic benefits require validation through pilot-scale amplification, this work has laid a low-cost, high-efficiency technical foundation for industrialization across three dimensions: raw material substitution, process optimization, and enzyme performance enhancement.

### Future perspectives and implications

The present study successfully establishes *P. oxalicum* UNN1 as an efficient cell factory for co-producing cellulases and xylanases using low-cost, waste-derived inducers and demonstrates its effectiveness in hydrolyzing agricultural residues. This integrated approach, encompassing strain selection, process optimization, and enzymatic valorization, provides a tangible pathway towards the sustainable and economical conversion of lignocellulosic biomass. Based on the present findings, future research will focus on two complementary tracks:

1. Mechanistic elucidation: The stable high-yield system established here offers an ideal platform for uncovering underlying molecular regulations. Subsequent comparative transcriptomics and proteomics analyses can elucidate the transcriptional network and secretory pathways responsible for the coordinated expression of cellulases and xylanases induced by the mixed carbon source (SBX + Avicel). Genetic engineering tools (e.g., CRISPR‑Cas9) could then be employed to functionally characterize key potential regulators (e.g., homologs of transcription factor CxrC), revealing the genetic basis for their high enzyme productivity. This knowledge will provide a theoretical foundation for rationally engineering the strain for further improvement.
2. Process integration and scale-up: To facilitate technology translation, pilot-scale efforts should focus on: (i) Using a statistical design of experiments (e.g. Plackett-Burman followed by RSM) to scale up and validate the economic viability and robustness of the low-cost fermentation process centered on pre-treated agricultural residues (e.g., bagasse, corn stover); (ii) developing formulation technologies for the crude enzyme cocktail to enhance its storage stability and handling convenience; and (iii) integrating this enzyme system with suitable mild pretreatment methods (e.g., steam explosion, deep eutectic solvent treatment) to overcome the hydrolysis bottleneck of lignin-rich raw materials like native sugarcane bagasse. The ultimate objective is to integrate these steps into a complete biorefinery chain: “pretreatment of agricultural waste → efficient enzyme production → targeted polysaccharide hydrolysis → manufacturing of high-value products (e.g., fermentable sugars, xylooligosaccharides).”

In summary, while providing a strain and a process scheme with clear industrial prospects, this study also builds a bridge connecting “applied optimization” with “basic mechanisms,” and linking “laboratory research” with “industrial piloting,” thereby contributing a novel solution for the valorization and sustainable utilization of lignocellulosic resources.

## Conclusions

In this study, a xylanase-overproducing fungal strain, *P. oxalicum* UNN1, was isolated, and its submerged fermentation (SmF) conditions were optimized. The strain demonstrated a competitive yield-stability profile compared to recent fungal platforms: it achieved high xylanase activities of (191.22 ± 4.27) U/mL (with SBX as the sole carbon source) and (142.32 ± 9.19) U/mL (with a mixture of SBX and Avicel), along with a FPase activity of (0.76 ± 0.03) U/mL. The crude enzyme exhibited optimal activity at pH 5.0 and 50 °C and demonstrated excellent stability—retaining over 75% cellulase activity and nearly full xylanase activity after 7 h of incubation under pH 4.0–6.0 (4 °C) and 40 °C (pH = 5.0) conditions. Notably, xylanase activity remained virtually unchanged even after 21 days of storage at pH 5.0 and 4 °C, with a half-life exceeding 72 h at 40 °C (pH = 5.5). In addition, Ca²⁺ and Cu²⁺ strongly suppressed both cellulase and xylanase activities. Finally, the crude enzyme efficiently hydrolyzed polysaccharide-rich agricultural residues such as citrus peel and corncob. Through single-factor and response surface optimization, the optimal hydrolysis conditions for SBX were determined as a xylanase loading of 480 U/g SBX, pH 5.5, and 40 °C, achieving a maximum reducing sugar yield of (0.355 ± 0.011) g/g SBX.

Collectively, this study confirms the significant potential of *P. oxalicum* UNN1 and its crude enzyme system in the enzymatic conversion of agricultural residues. By employing low-cost agricultural residues as an inducer for efficient enzyme production and coupling it with an optimized hydrolysis process, this work outlines a technical route with clear potential for cost advantages for agricultural residue valorization. Nevertheless, to realize its large-scale “high-value utilization” and “high-stability saccharification,” further pilot-scale validation, detailed cost analysis, and precise product profiling are required. The findings presented here establish a necessary experimental foundation for the continued development and application in this direction.

## Electronic Supplementary Material

Below is the link to the electronic supplementary material.

Supplementary Material 1

## Data Availability

Data will be made available on request.
