# Supplementary material for: Optimization of fermentation conditions for cellulase/xylanase production and hydrolysis conditions for efficient conversion of agricultural residues using Penicillium oxalicum UNN1
Source: Bioresour Bioprocess. 2026 Mar 26;13(1):39. doi: 10.1186/s40643-026-01035-2 (PMC13022107; doi:10.1186/s40643-026-01035-2)
Supplement: Supplementary file 1 — Supplementary Material 1 [file 40643_2026_1035_MOESM1_ESM.docx]

**Supplementary Information**

**
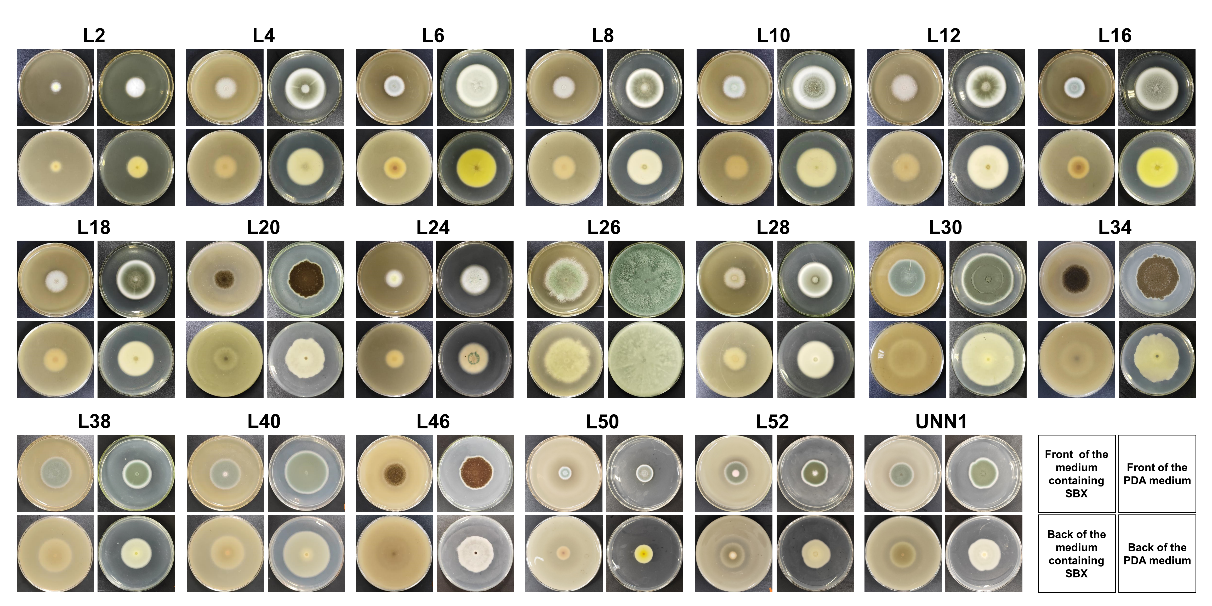
**

**Fig. S1 Plate phenotypes of 20 xylanase-active strains.** In this experiment, 2 μL of pure fungal spore suspension was inoculated onto a solid medium, followed by incubation at 28°C for 5 days before photographing and observation. SBX: sugarcane bagasse xylan (alkaline-extracted). PDA: potato dextrose agar
